# The New York Institute of Stomatology

**Published:** 1899-04

**Authors:** 

**Affiliations:** The New York Institute of Stomatology


### THE NEW YORK INSTITUTE OF STOMATOLOGY.

A meeting of the Institute was held on the evening of Tuesday, January 3, 1899, at the residence of Dr. S. E. Davenport, No. 51 West Forty-seventh Street, the President, Dr. E. A. Bogue, in the chair.

The minutes of the previous meeting were read and approved.

### COMMUNICATIONS ON THEORY AND PRACTICE.

*Dr. E. T. Payne.*—When I received an invitation from your secretary, it occurred to me that a case of facial neuralgia which I had not long ago might be of interest to the Institute. The pain had been coming on for two years, and the case had been given over as hopeless by the local physicians. When it came under my treatment I found the cause and effected a cure, which seemed such a revelation that I was invited to lay the case before the medical society in Western Connecticut somewhat in detail.

I found that the medical gentlemen were quite in the dark concerning the cases of facial neuralgia which result from a secondary growth in the pulp-cavities of teeth.

The subject of neuralgia is so wide that a paper would be insufficient to do it justice. I shall, therefore, not undertake to refer to the disease in general, but only as applied to one form of facial neuralgia, and to indicate what is sometimes the cause. To you gentlemen who are so familiar with the malady I need go into very few details. I will simply say that the abnormal growth known as pulp-stone occasionally obtains in the pulp-cavities of the teeth and causes great suffering. It is difficult for one who has not had considerable experience to locate the particular tooth and give relief. The case was that of a lady of thirty-five. One of the superior molars was somewhat troublesome about one year ago, and she wished me particularly to examine that. I did so and found it apparently in perfect condition. At that time she said nothing about neuralgic pains. About two months ago she called and said she was suffering intensely, and wanted another examination of her teeth; that her doctor was unable to do anything to relieve her. The most severe pain seemed to centre under the orbit, but the darting, throbbing pains were diffused over the entire right side of the face.

My mind reverted to the previous visit, and I concluded to apply the usual tests for pulp-stone. I located the trouble in a molar, opened to the pulp, and applied the usual preparation to destroy it. After thirty-six hours I removed the medicament and cleansed the cavity. After four days I opened into the pulp-cavity and found the specimen which I bring for your inspection.

I removed the pulp from the roots and filled them just as I would when an exposed pulp had been devitalized. Complete relief resulted.

To diagnose pulp-stone is not very difficult:

1. Such exertion as will increase the action of the heart will aggravate the trouble.

2. Sounding the tooth with a steel instrument will give the same result as incipient periostitis.

3. Sudden closing of the teeth indicates periostitis.

4. Closing the teeth gently and biting ever so hard is not painful, and proves that periostitis is absent.

5. Lateral pressure does not give pain, as would be the case with periostitis.

You will note three positive tests and two which may be called negative.

Dentists will do well to call the attention of their medical friends to this matter. More cases of neuralgia result from trouble with the teeth than many suppose.

*The President.*—I suppose Dr. Payne considers that when the pulp-stone has been in existence as long as this one, that there is a certain amount of inflammatory action in the pulp.

*Dr. Payne.*—Of course, the disturbance resulting from the pressure of the foreign substance caused at least momentary pulpitis, but acute pulpitis did not obtain, I am quite sure, or else strangulation would have resulted during the weeks and months the patient had been suffering.

*Dr. S. H. McNaughton.*—I had a somewhat similar case about three years ago. The patient, a man of fifty-five years of age, was brought to me for consultation by his dentist. He had suffered much from neuralgia of the left side of the face and head, and had been treated by his dentist, by a consulting oral surgeon, and by his physician for a month or more. There had been an unusually severe attack the previous day. Examination showed that all the upper teeth had long since been removed, and that all the teeth in the lower jaw, with the exception of the second molar, were devitalized. The roots of the incisors, left cuspid, and I think those of the left bicuspids had been amputated. The wound, although not healed, had a healthy appearance.

My first thought was that such pain, if caused by a tooth, was due to one with an irritated pulp, and the second molar was the only tooth on the left side of the lower jaw which contained a live pulp. Rubber dam was applied and a stream of hot water was played on the tooth for two or three minutes without any sensation. Shortly after removing the dam the patient said that I had started the pain.

Cold water for a minute or two gave relief. In reply to questions the patient said that the pain often began during a meal, after exertion, and in the evening. The tooth was drilled to the pulp-chamber and arsenous acid applied. His dentist resumed the treatment, and I was informed that there had been no return of the pain after the application of arsenic, and that the pulp and several pulp-stones had been removed.

The point which I wish to make is, that the application of heat should be made for two or three minutes or more instead of but for an instant. I have had several similar cases where the application of heat for a few minutes has been a great aid in establishing a diagnosis.

*Dr. J. F. P. Hodson.*—In response to your invitation, Mr. President, I have to say that it so happens that I am in deep tribulation in this direction at the present time. Some months ago a young Scotchwoman, visiting this country, suddenly became practically blind in one eye, and both her physician and her oculist, having surely defined the normality of her system, and come, by the process of exclusion, to define the teeth as the cause, turned to me for dental assistance.

I have thus far been wholly unable to render any, although I am confident that I shall eventually find the cause in the teeth, as they are enormously filled, whole crowns and many of the teeth being built up with amalgam. I suspect pulp-stones, but she has not had the slightest pain in any of the teeth, and I can get no response whatever to any of the usual or unusual tests which I am making.

There are nine or ten teeth, any one or more of which might be the culprit, but I have never in all my experience met with any case where some slight leading could not be had, either by there being but one or two suspicious ones, or by some slight response somewhere to the array of tests to differentiate the many.

*Dr. S. C. G. Watkins.*—Dr. William P. Cooke, of Boston, read a valuable paper on this subject before the Academy of Dental Science, in Boston, May 7, 1890, the title of which was "Formations in the Pulp-Cavity." It was published in the INTERNATIONAL DENTAL JOURNAL of December, 1890. He gave stereopticon views to illustrate his paper, showing how many cases there were of pulp-stones, calcified tissue in the pulp, and calcified tissue connected to the side of the pulp-cavities and chambers. I think that, of five thousand teeth which he examined, a great proportion of them had calcified tissue in some form or other, either in the form of pulp-stone or spicula. He projected a great number of pictures on canvas at that time, showing conclusively his results and his beautiful work along that line. I think it would be well for the members to look that paper over again; it would be most interesting to all of us, for certainly a great many of those cases are overlooked. We find some, but I think there are many which we do not find and perhaps do not suspect, yet they cause a great deal of trouble. I am very glad to hear the remarks by Dr. Payne on this subject; they are exceedingly interesting, and I feel that I have learned something.

*Dr. C. O. Kimball.*—I wish to speak for a few moments of a very simple appliance which I have used many years for the purpose of holding back the gum from cervical cavities. As the time is limited, I will go directly to the explanation without any preamble, except this, that we all feel the difficulty in those cases in which the cavity runs around the neck of the tooth close to the gum, and especially where, by the receding of the gum, the sulcus between the roots of molars is partly exposed.

For all these cases I use hickory sticks shaped to fit the tooth and cavity, and held in position with the left hand, so that the rubber dam is held back with it.

I have tried various kinds of wood but have come to this for these reasons: Hickory has a certain toughness of fibre that hardly any other wood possesses; it can be moulded exactly to the tooth and to the cavity as well, even in some cases bent to a curve, and yet it is stiff, so that it does not give, and has a fine fibre, so that it can be made thin without splitting. I should have hesitated in calling attention to so simple a device as this, but happening to speak of it to one or two members who have had a very large experience, I was surprised to find the idea rather novel to them. Hence it seemed to me wise to bring it forward. I have here some of the sticks that I use right along in practice, and the shapes that they naturally take can be seen. I gouge out the upper surface of the stick until it fits the margin or follows the line of the cavity. I then cut the end of the stick so that it fits the neck of the tooth, shaving the back of it down to a thin knife edge; in this way the stick is adapted to each individual case, which is the one great advantage of the stick over a steel instrument. For instance, on a lower molar, where the gums have receded and the cavity has a double-U curve or line at the margin of the gum, running down on each root and up towards the middle, one of these sticks can be exactly adapted to each case, even having a point which juts into the space between the roots, not only holding the dam in place, but covering and compressing the gum between the roots where the dam cannot go. Another advantage of using the stick is, that we can hold down the gum and dam firmly, without pressing with undue force on any one part, but restraining it all with equal pressure. As one gets in the habit of doing this the sticks can be fitted very quickly and readily.

*Dr. Hodson.*—When Dr. Kimball did me the honor to ask me to speak on this subject, and to exhibit some of my own appliances, I assured him that what I could have to say would amount to very little, but perhaps I cannot do better than tell you of my own experience in the premises. First, in respect to the hickory stick, I should feel that they would lack in positiveness, in that unyielding exactness of thin edge characteristic only of metal. It would seem to me, also, far more bulky than the delicate metal edge which takes up no appreciable room, and so would cover and hide the very under-the-gum cervical edge which I had probably been at much pains to uncover. I should presume, moreover, that the stick would present a little rope-like or turned-over edge that would destroy the delicacy of my marginal exposure. Worse than all, it would absorb and conduct to the gold the moisture which all my endeavors had been directed to exclude. In a word, if I needed to hold the gum back when the cavity ran underneath it, it would require an *absolute* holding. If, on the other hand, there remained an isthmus of material between the cavity margin and the gum line sufficient to accommodate anything so bulky as the wood, I would not need anything. Thirty years ago—in 1870, I think—I wrote a paper on the rubber dam, and directions for its application, and among the instruments, appliances, and clamps, which I then suggested, was this type of small thin chisels, with the edge concave to fit the rotundity of the neck of the tooth. They are of various shapes,—straight, hatchet-shaped, etc.,—to accord with the different positions in which they would be held for different teeth. They are sharp and thin, and are intended to be placed by gently carrying the edge of the rubber (I use the thinnest dam for all purposes) above the cervical margin with a small flat burnisher, and while so holding it place the appropriate instrument in position, holding it firmly and positively against the tooth (*not* against the gum) throughout the operation. These I show might be handsomer,—they would even be the better for wooden handles, for instance,—but they have seen much service, as, though changed in shape or in angle very often for individual cases, they are the identical ones I have used daily for all these years.

I am accustomed always to get as full an exposure of the cervical margins as possible before operating, by packing a bit of gutta-percha—with its surface moistened, after warming, with oil of cajuput to make it stick—into the cavity and against the gum for a day or two. The gutta-percha may be made to stay in place, if other methods are inadequate, by tying thin floss silk around the tooth and over the filling. It is sometimes expedient, in case of any considerable extension of the cavity *around* the tooth, to fill it in two sections, by placing hard gutta-percha, or oxyphosphate, in half the cavity to prevent leakage, filling the empty portion complete; then shifting the holder to the portion filled with gutta-percha and filling that.

Two of these little instruments are right and left, and hold down the dam upon the posterior surface of partially developed back teeth, as, this posterior edge being held, the retention of the dam in *such* cases is sure. Tipped-forward teeth, with their totally different presentation, would require totally different treatment. These instruments seem to me far better and more gentle in such cases than clamps, and, indeed, it is a curious circumstance that, although I had the pleasure, in the article in 1890 previously referred to, of introducing the idea and system of clamps, which has now grown to such beautiful applications, I have never found occasion or necessity for their employment since that time.

I am very thankful to Dr. Kimball for his suggestion in respect to the wood. I have used it somewhat during the two or three weeks since he mentioned it to me, and think that for certain cases, as I get accustomed to it, I shall like it better.

*Dr. J. B. Hawes.*—I do not know that I can add anything to what has already been said. I always rely upon the rubber dam in filling such cavities, which can be easily kept in place by an instrument holding up a fine silken ligature, not tied so tight as to prevent it from adapting itself to the gum line, and with the knot always on the side of the tooth opposite to the cavity.

*Dr. F. Milton Smith.*—The subject has narrowed down, as I understand it, to the keeping of cervical cavities dry. I did not so understand the announcement, and had other thoughts in mind. The suggestions that have been made have been helpful to me, for I have been interested in this class of cavities for many years. About ten years ago there was advertised by the S. S. White Company what was known as the Howe cervical clamp, with which all are probably familiar. At that time it was the best clamp for the purpose that I could find in any of the dental depots. I purchased it, and was greatly disappointed to find that it did not do the work I desired, and it seemed to me that something adjustable was needed for the purpose. One night when I could not sleep the idea occurred to me, Why not make such a clamp and make it adjustable? I got up the next morning and made this clamp, which, so far as I know, is the first adjustable cervical clamp ever given to the public; but I found after making and using it and offering it to a certain company to manufacture for the profession that already the same clamp had been patented and stowed away until after the market had been supplied with the one first referred to. That clamp which was patented is the Howe adjustable clamp, and was the first adjustable clamp patented. I am glad that I found out what is done with useful patents after they are taken out.

My usual plan for keeping cervical cavities dry is to tie a silk ligature around the neck of the tooth. Where it is much higher on the labial side, I hold the ligature above the cavity with a hooked instrument held in the left hand. In many cases I dispense with the ligature, depending upon steel instruments to hold the dam above the cavity.

*Dr. L. C. Leroy.*—Dr. Smith's description of the silk ligature calls to mind the steel wire, that can be conveyed about all teeth and be held nicely in place without the application of any other instrument. I have found it of excellent service, and it is so useful that it should not be lost sight of in a discussion of this kind. It is not a clamp, but a piece of soft "binding" wire simply twisted about the tooth in the way that we twist any wire, and it will remain in the position in which it is wound.

*Dr. Hawes.*—In the preparation of the cavity, I take great pains to make it of such shape that after it is one-third filled, the gold will hold itself in position and the overhanging gold will hold the rubber dam.

Dr. Dwight L. Hubbard presented a paper on "Vocal Hinderances."

(For Dr. Hubbard's paper, see page 213.)

DISCUSSION.

*The President.*—You have heard this very interesting paper, and I hope it will not be allowed to pass without proper discussion. Dr. Hubbard did not go into all the points, but he has done enough to serve as a starting-point.

*Dr. R. H. M. Dawbarn.*—There are some points which come to mind under the head of vocal hinderances which were not mentioned by Dr. Hubbard,—lymphoid growths in the pharyngeal vault, with their associated phenomena; certain irregularities of the teeth which render the proper enunciation of the labio-dentals (*f* and *v*) impossible.

Concerning the lymphatic supply of the nasopharynx, it would not be amiss to speak of Waldeyer's tonsillar ring, comprising which from above downward are Luschka's tonsil, the two tubal tonsils, two faucial tonsils, and the glossic tonsil.

*Dr. Hubbard.*—My attempt in writing this paper was to generalize and submit matters of general interest suggestively. Of course, it would be impossible to exhaust such a broad field, for a whole library would be the result of such an attempt. I am obliged to Dr. Dawbarn for referring to lymphoid growths in the pharyngeal vault. I fully recognize their importance. I have, upon former occasions, said so much upon this subject that I thought it well not to bear upon it now.

*Dr. John I. Hart.*—I am very glad to have listened to Dr. Hubbard's excellent paper, and I think that it has a practical character which is of interest to us all. A proper consideration of the paper would necessitate its division into two headings, one academic and the other practical. I am glad that Dr. Hubbard endorses those of us who have recognized the necessity for proper hygiene of the mouth; and it will lead those who have not given the subject much thought to the necessity of taking or causing their patients to take greater care of the mouth from a hygienic stand-point.

*The President.*—It would have been a source of pleasure, I think, as does Dr. Dawbarn, if the essayist had led us a little farther in the consideration of the question of phonation and all that is concerned in it.

This would, I am aware, have brought in cleft palate, the saddle-shaped jaw, irregularities of the dental arches, adenoid or lymphoid growths, and a consideration of the question whether irregularities of the teeth do not frequently produce these bodies in the posterior nares. On the other hand, whether the presence of adenoid growths does not conduce to such irregularities of the upper jaws as lead to irregularities in the positions of the teeth and to such changes in the vault of the palate that accurate normal phonation is impossible.

I am sure we all feel that the essayist has but opened the question this evening.

*Dr. J. Morgan Howe.*—I move that a vote of thanks be extended to Dr. Hubbard for his very interesting and able paper.

Carried.

Adjourned.

F. L. Bogue, M.D., D.D.S.,
*Editor The New York Institute of Stomatology.*

